# Unravelling the Antifungal Effect of Red Thyme Oil (*Thymus vulgaris* L.) Compounds in Vapor Phase

**DOI:** 10.3390/molecules25204761

**Published:** 2020-10-16

**Authors:** Loris Pinto, Maria Addolorata Bonifacio, Elvira De Giglio, Stefania Cometa, Antonio F. Logrieco, Federico Baruzzi

**Affiliations:** 1 Institute of Sciences of Food Production, National Research Council of Italy, Via G. Amendola 122/O, 70126 Bari, Italy; antonio.logrieco@ispa.cnr.it (A.F.L.); federico.baruzzi@ispa.cnr.it (F.B.); 2 Department of Chemistry, University of Bari, Via Orabona, 4, 70126 Bari, Italy; maria.bonifacio@uniba.it (M.A.B.); elvira.degiglio@uniba.it (E.D.G.); 3 Jaber Innovation S.r.l., Via Calcutta 8, 00144 Rome, Italy; stefania.cometa@jaber.it

**Keywords:** essential oils, GC-MS analysis, antifungal activity, synergistic activity, vapor phase

## Abstract

The aim of this work was to evaluate the antifungal activity in vapor phase of thymol, p-cymene, and γ-terpinene, the red thyme essential oil compounds (RTOCs). The Minimum Inhibitory Concentration (MIC) of RTOCs was determined against postharvest spoilage fungi of the genera *Botrytis*, *Penicillium*, *Alternaria*, and *Monilinia*, by measuring the reduction of the fungal biomass after exposure for 72 h at 25 °C. Thymol showed the lowest MIC (7.0 µg/L), followed by γ-terpinene (28.4 µg/L) and p-cymene (40.0 µg/L). In the case of *P. digitatum* ITEM 9569, resistant to commercial RTO, a better evaluation of interactions among RTOCs was performed using the checkerboard assay and the calculation of the Fractional Inhibitory Concentration Index (FICI). During incubation, changes in the RTOCs concentration were measured by GC-MS analysis. A synergistic effect between thymol (0.013 ± 0.003 L/L) and γ-terpinene (0.990 ± 0.030 L/L) (FICI = 0.50) in binary combinations, and between p-cymene (0.700 ± 0.010 L/L) and γ-terpinene (0.290 ± 0.010 L/L) in presence of thymol (0.008 ± 0.001 L/L) (FICI = 0.19), in ternary combinations was found. The synergistic effect against the strain *P. digitatum* ITEM 9569 suggests that different combinations among RTOCs could be defined to control fungal strains causing different food spoilage phenomena.

## 1. Introduction

Essential oils (EOs), mixtures of several compounds extracted from vegetable tissues, are characterized by a variety of biological activities, including antimicrobial, antioxidant, and insecticidal effects [1,2]. EOs have recently gained attention as eco-friendly pesticides and as an alternative to the use of synthetic fungicides that pose threats related to food residues, fungal resistance, and negative environmental impact [3,4]. Their antimicrobial action depends on the chemical composition, the concentration of each active compound in the EO, and their ability to be released and to reach the microbial target, as well as the sensitivity of microbial strains [4,5]. Numerous studies have shown that EOs composed by different monoterpenes, monoterpenoids, sesquiterpenes, and other volatiles (esters, ketones, aromatic phenols, alcohols, aldehydes, ethers, hydrocarbons, coumarins, and organic acids) effectively reduced the development of postharvest spoilage fungi of the genera *Alternaria*, *Botrytis*, *Aspergillus*, *Fusarium*, *Penicillium*, and *Rizophus* [6]. Interestingly, different studies demonstrated that EOs have a better antifungal effect in vapor phase rather than in liquid phase [7,8]. However, few studies identified the chemical compounds responsible for microbial inhibition/inactivation [9]. Although several studies reported the antifungal action of the main essential oil compounds (EOCs), such as thymol, carvacrol, p-cymene, cinnamaldehyde, eugenol, and citral, by direct contact assays [10,11,12,13], few studies focused on the EOCs antimicrobial action in vapor phase. The proportion of single compounds in the liquid phase is relatively stable, but volatile compounds, introduced in non-saturated environment, start to diffuse at different rates until they reach the equilibrium [9]. Therefore, their concentration in vapor phase could be different from that measured in the EO. For these reasons, the antimicrobial action detected by direct contact assay may differ from that in vapor phase.

Božik et al. (2017) found that thyme oil vapors effectively controlled *Aspergillus* spp. growth on oat, but this treatment negatively affected the sensory profile of raw and cooked samples [14]. Conversely, EO vapors application using warm air flow demonstrated to reduce negative sensory properties of EOs, without affecting their antifungal activity [15]. In regard to the antifungal activity of volatile EOCs, Tang et al. (2018) demonstrated that geraniol and citral inhibited *A. flavus* and *A. ochraceus* growth [16], whereas thymol negatively affected fungal spore viability and surface mycelium structures of *Monilinia fructicola* [17].

The use of different combinations of EOCs or essential oils showed interactive antimicrobial effects, as demonstrated for direct contact assays against fungi, yeasts, and bacteria [18,19]. The recent work carried out by OuYang et al. (2020) showed synergistic activity of cinnamaldehyde and citronellal in reducing the mycelium growth of *P. digitatum* by direct contact. The combination of these EOCs sped up the damage of the cell wall and the cell membrane [20]. It is interesting to note that the fractionation of the *Cymbopogon citratus*, *Ocimum gratissimum*, and *Thymus vulgaris* EOs highlighted synergistic activities among non-active fractions against *P. expansum*, probably caused by the interactions between oxygenated and terpene hydrocarbons [21].

Recently, synergistic interactions have been also found for mustard and cinnamon essential oil vapors against post-harvest spoilage molds of the genera *Aspergillus*, *Botrytis*, *Fusarium*, *Geotrichum*, *Penicillium*, and *Rizophus* [22]. Triple combination of cinnamon bark, citronella and may change essential oil vapors showed synergistic effects against *P. corylophilum* [23], whereas the binary combination of thyme and oregano EOs vapors showed additive or synergistic effects against *P. expansum*, depending on their ratios [24]. It is difficult to reveal antifungal interactions among EOCs using complex mixtures, such as commercial EOs. Our previous work showed that red thyme oil (RTO) vapors controlled the external mycelium growth of *Penicillium* spp. strains on oranges. In particular, the mycelium growth was reduced by 66–70%, on average, during 16 days of cold storage; however, it was not possible to determine which EOC mainly contributed to the antifungal action, and if interactive effects among EOCs occurred [5]. Therefore, a deeper investigation on the antifungal action of combinations composed by pure EOCs in vapor phase is necessary.

RTO is one of the most active essential oil against postharvest spoilage fungi [5,25], and its chemical composition includes monoterpene hydrocarbons, oxygenated monoterpenes, sesquiterpene hydrocarbons, and oxygenated sesquiterpenes at different concentrations [26]. Thymol, p-cymene, and γ-terpinene, the three main compounds present in the EO used in this work, are Generally Recognized as Safe (GRAS) [27].

The antifungal action of RTO compounds (RTOCs) in vapor phase has not yet been investigated and the interactive effects among EOCs against fungi need further evaluations. In this work, the antifungal activity of each RTOC was firstly predicted in silico in order to highlight their biological effects and mechanisms of action. The antimicrobial action of RTOCs vapors was further assessed against postharvest spoilage fungi. Then, interactive effects among individual compounds were assessed using binary and ternary combinations. The concentration of active molecules was followed in vapor phase trials by means of gas-chromatographic analyses.

## 2. Results

**Figure 1:**
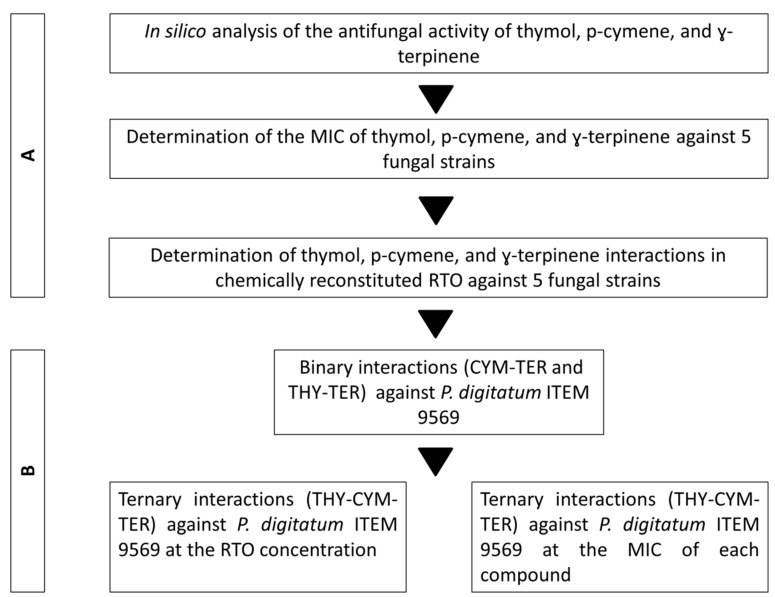
Workflow of the experimental activity. Evaluation of the antifungal effect of single red thyme essential oil compounds (RTOCs) (**A**) and evaluation of their potential additive/synergistic effect against the RTO-resistant *P. digitatum* ITEM 9569 (**B**). Abbreviations: THY, thymol; CYM, p-cymene; TER, γ-terpinene.

The experimental activity carried out in this work is depicted in Figure 1. Firstly, the evaluation of the antifungal effect of single RTOCs was performed against 5 fungal strains (Figure 1A). Then, potential additive/synergistic effect of RTOCs was assessed against the RTO resistant *P. digitatum* ITEM 9569 (Figure 1B).

### 2.1. Evaluation of the Antifungal Effect of RTOCs

#### 2.1.1. In Silico Analysis

The identified RTOCs were subjected to the Prediction of Activity Spectra for Substances (PASS) online tool in order to predict their potential antimicrobial activities and suggested targets. These analyses are based on quantitative structure−activity relationship linear (QSAR) models. Membrane integrity antagonist, membrane permeability enhancer, general pump inhibitor, antifungal, steroid synthesis inhibitor, and oxidizing agent descriptors were used to indicate biological activity (Table 1).

Cell wall synthesis inhibitor, DNA synthesis inhibitor, protein synthesis inhibitor, lanosterol 14 alpha demethylase (CYP51A1) inhibitor, and squalene epoxidase inhibitor descriptors were used to indicate the mechanism of action. Moderate antifungal activity was predicted for the three RTOCs. High potential as membrane integrity antagonist and membrane permeability enhancer was predicted for thymol and p-cymene. Biological effects with high potential (Pa − Pi ≥ 0.5) for γ-terpinene were related to the membrane integrity antagonism and the general pump inhibition (Table 1). Other biological effects showed low potential (Pa − Pi ≤ 0.2). Potential mechanisms of antifungal action, such as the lanosterol 14 alpha demethylase and the squalene epoxidase inhibition, were predicted with low potential for the three RTOCs. The prediction of potential biological effects produced by the RTOCs focused mainly on membrane damages or in the reduction of its functionality. However, the biological effects showing low Pa values could enhance the damages of the cell membrane, contributing to the RTO antifungal activity. Therefore, thymol, p-cymene and γ-terpinene vapor contact assays were carried out to verify the occurrence of additive and/or synergistic antifungal effects.

#### 2.1.2. Antifungal Activity of RTOCs

The minimum inhibitory concentration (MIC) produced by thymol, p-cymene, and γ-terpinene by vapor assays against five fungal strains is reported in Table 2.

The vapor assays where carried out in triplicates for each strain, showing differences in mycelium dry weight within the range of ca. 10–15%. As a consequence of this experimental result, we defined the MIC as that concentration able to produce, in comparison to control plates, an average reduction in fungal dry biomass higher than 20%. The mean dry mycelium weight of control samples resulted to be 75, 32, 81, 49, and 138 mg for *P. digitatum*, *P. italicum*, *B. cinerea*, *M. laxa*, and *A. alternata*, respectively. The concentration of each RTOC significantly affected (*p* ≤ 0.05) the fungal biomass of all strains. The mean MIC value was 7.0 ± 5.6 µg/L, 40.0 ± 28.3 µg/L, and 28.4 ± 11.3 µg/L for thymol, p-cymene, and γ-terpinene, respectively (Table S1, Supplementary Results). Thymol reduced the fungal biomass of all strains, whereas p-cymene and γ-terpinene were not active at the highest concentration against *A. alternata* ITEM 4215. The mean percentage of reduction in fungal biomass at the MIC level was 68.1 ± 27.3%, 68.9 ± 36.7%, and 85.0 ± 18.3% for thymol, p-cymene, and γ-terpinene, respectively. *A. alternata* was the most sensitive strain to thymol exposure, whereas *M. laxa* was the most resistant; *P. italicum* and *B. cinerea* were the most sensitive strains to γ-terpinene treatment, whereas *M. laxa* was the most sensitive top-cymene exposure. *P. digitatum* and *B. cinerea* were the most resistant strains to γ-terpinene and p-cymene vapor exposure, respectively (Table 2; Table S1). Among the three RTOCs, thymol was the most active against fungal strains, showing the lowest MIC value and a broad spectrum of antifungal action (Table S1, Supplementary Results).

The commercial RTO concentration inhibiting all fungal strains was 66.6 µL/L (Table 2); at the beginning of incubation, this volume of EO released 0.07% of thymol, 66.7% of p-cymene, and 27.1% of γ-terpinene in the plastic box. These concentrations remained almost stable showing values of 0.03% of thymol, 71.9% of p-cymene, and 24.5% of γ-terpinene, after 72 h of incubation at 25 °C, as already reported [5].

#### 2.1.3. Interactions among RTOCs

In order to evaluate the antifungal activity of each RTOC, the vapor assays started considering the composition of commercial RTO (Figure S2): 43.4 ± 4% thymol, 37.5 ± 3% p-cymene, and 19.1 ± 2% γ-terpinene. Since the common MIC of commercial RTO for all fungal strains considered in this work was 66.6 µL/L (Table 2), the relative concentrations of RTOCs in the chemically reconstituted RTO were defined as 25.7 µg/L thymol, 20.0 µg/L p-cymene, and 11.4 µg/L γ-terpinene.

At the end of incubation, 66.6 µL/L of reconstituted RTO caused the complete absence of mycelium growth (100% inhibition) for all fungal strains (Table 3); after 72 h of incubation at 25 °C, 0.80 ± 0.18% thymol, 70.2 ± 1.3% p-cymene, and 29.0 ± 0.8% γ-terpinene were found. These values were close to that found at the beginning of incubation (0.6 ± 0.2% thymol, 71.1 ± 0.8% p-cymene, and 28.3 ± 1.2% γ-terpinene). These results suggest that the ratio among RTOCs, as well as their active concentration, are not significantly modified in the in vitro system employed in this work. Even though small differences were found in the percentage of RTOCs in vapor phase, similar release kinetics were found between reconstituted and commercial RTO.

The removal of p-cymene (20.0 µg/L) from the vapor phase (combination 1, thymol-γ-terpinene (THY-TER)) did not result in any loss of the antifungal activity, suggesting the absence of a strong antagonism of this compound in the RTO at 20.0 µg/L. On the contrary, the removal of γ-terpinene (11.4 µg/L) from the RTO mixture (combination 2, THY-p-cymene (CYM)) determined a reduction in the antifungal activity in comparison to THY-CYM-TER and THY-CYM combinations only for *P. digitatum.*

The removal of thymol from RTO (combination 3, TER-CYM) did not produce inhibition of *P. italicum* and *A. alternata* growth. A strong reduction in the antifungal activity was found against *B. cinerea* and *M. laxa* in comparison to the reconstituted RTO, whereas only *P. digitatum* still showed high inhibition of fungal growth.

In the case of *P. italicum*, *B. cinerea*, *A. alternata*, and *M. laxa*, the growth inhibition determined by thymol exposure did not change with the addition of p-cymene, γ-terpinene, nor both compounds (Table 3). Conversely, thymol alone determined the lowest reduction in the *P. digitatum* ITEM 9569 fungal biomass (62%) in comparison to THY-CYM (73%), THY-TER (100%), and THY-CYM-TER (100%) combinations and in comparison to other fungal strains (Table 3).

However, in the case of *P. digitatum* ITEM 9569, two combinations showed potential interactive effects in comparison to the single RTO compound exposure. Indeed, the exposure to the CYM-TER combination determined a significant enhancement of the antifungal activity compared to CYM or TER application. Likewise, the THY-TER combination determined higher reduction of the fungal biomass than single THY or TER exposure (Table 3). Since selected RTOC combinations seem to exert higher antifungal activity than single compounds only for this strain, further antifungal assays were carried out to shed light on potential antifungal interactions among these compounds.

### 2.2. Interactions between RTOCs against P. digitatum ITEM 9569

#### 2.2.1. Binary Combinations

In our previous work, *P. digitatum* ITEM 9569 was less sensitive to RTO vapor exposure in comparison with *P. italicum* ITEM 9571 [5]. In this work, *P. digitatum* ITEM 9569 showed the highest resistance to thymol (25.7 µg/L, Table 3), even though binary RTOCs combinations determined higher fungal biomass reduction than single compounds (Table 3). In order to understand the specific antifungal activity of selected RTOCs against *P. digitatum* ITEM 9569, the antifungal effect of CYM-TER and THY-TER mixtures was evaluated using a modified checkerboard assay [28]. These two binary combinations (CYM-TER and THY-TER) were selected because they determined a reduction of the *P. digitatum* fungal biomass significantly (*p* ≤ 0.05) higher than that caused by the single compounds (Table 3). The MIC of thymol, p-cymene, and γ-terpinene in the checkerboard assay resulted to be 6.4 µg/L, 40.0 µg/L, and 45.5 µg/L, respectively. The MIC of γ-terpinene and p-cymene in the CYM-TER combination (22.8 µg/L for γ-terpinene and 20.0 µg/L for p-cymene) was equal to 1/2 MIC of each compound tested alone.

**Figure 2:**
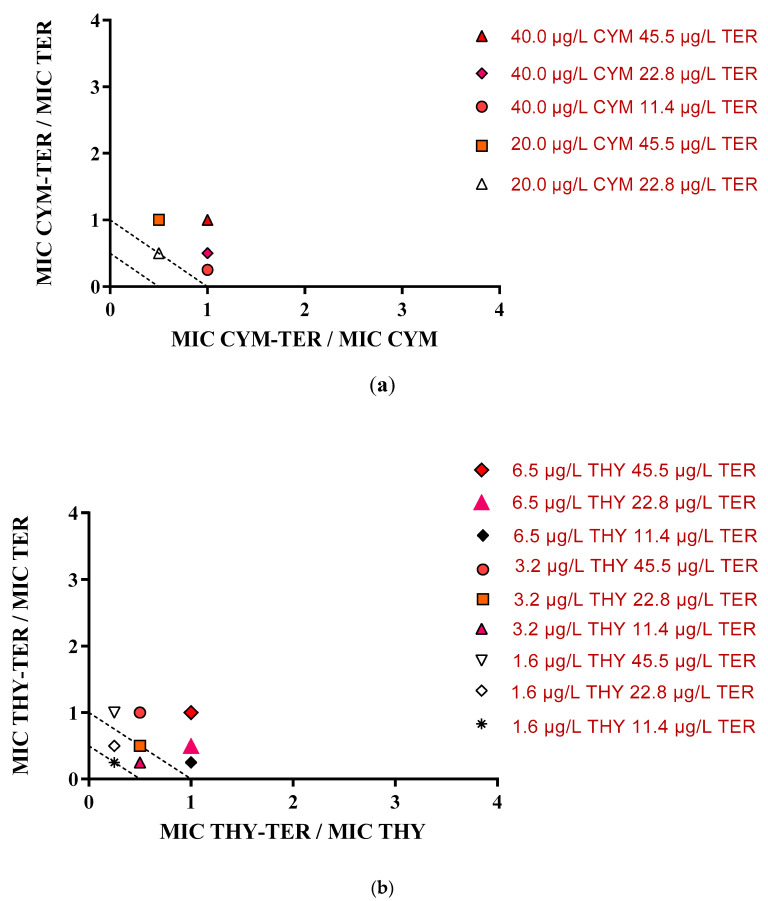
(**a**) Isobologram of p-cymene and γ-terpinene in different ratios against *P. digitatum* ITEM 9569; (**b**) isobologram of thymol and γ-terpinene in different ratios against *P. digitatum* ITEM 9569. In this case, the fractional inhibitory concentration (FIC) values are reported on each axis.

Isobolograms were used to classify the RTOC interactions. The points below or on the 0.5:0.5 line were interpreted as synergistic, the points between the 0.5:0.5 and 1.0:1.0 line were interpreted as additive, whereas the points between the 1.0:1.0 line and 4.0:4.0 line were classified as non-interactive in accordance with Suliman et al. (2010) [28]. As shown in the isobologram reported in Figure 2A, the interactive effect determined by the CYM-TER combination resulted additive (white triangle, Fractional Inhibitory Concentration Index (FICI) = 0.5). GC-MS analysis (representative chromatograms in Figure S3) revealed that this combination (Figure S3D) produced 0.44 ± 0.01 L/L of γ-terpinene (44.5 ± 1.2%), and 0.56 ± 0.02 L/L of p-cymene (55.5 ± 1.9%). The other CYM-TER combinations produced non-interactive effects (1≤ FICI ≤ 4, other points in the Figure 2A). It is interesting to note that the MIC of p-cymene was not achieved with the addition of 5.7 or 11.4 µg/L of γ-terpinene. As concerns γ-terpinene, MIC value was not achieved with the addition of 5.0 µg/L of p-cymene. These negative interactions could be related to a different volatility of RTOCs in binary combinations. Indeed, the vapor pressure of p-cymene is higher than that of γ-terpinene and thymol [29], and some combinations could result in a different volatility of the RTOCs, reducing their concentration in vapor phase and then their antifungal activity.

Conversely, the MIC of thymol and γ-terpinene in the THY-TER combination (1.6 µg/L for thymol and 11.4 µg/L for γ-terpinene) was equal to 1/4 MIC of each compound. It is interesting to note that TER at 11.4 µg/L and THY at 1.6 µg/L did not show antifungal activity (Table 3 and Table S1), whereas their combination produced a slight synergistic effect (FICI = 0.5) (asterisk in Figure 2B on the 0.5:0.5 line). GC-MS analysis (chromatogram C in Figure S3) revealed that this THY-TER combination produced 0.013 ± 0.003 L/L of thymol (1.3 ± 0.3%), and 0.99 ± 0.03 L/L of γ-terpinene (98.7 ± 2.8%). Three THY-TER combinations (1.6 µg/L THY and 22.8 µg/L TER, 3.2 µg/L THY and 11.4 µg/L TER, 3.2 µg/L THY and 22.8 µg/L TER) determined additive effects (0.5 ≤ FICI ≤ 1, points between the 0.5:0.5 line and the 1:1 line). The other THY-TER combinations, with different concentration of THY or TER, determined non-interactive effects (1≤ FICI ≤ 4, points above the 1:1 line in Figure 2B).

#### 2.2.2. Ternary Combinations Interactions

**Figure 3:**
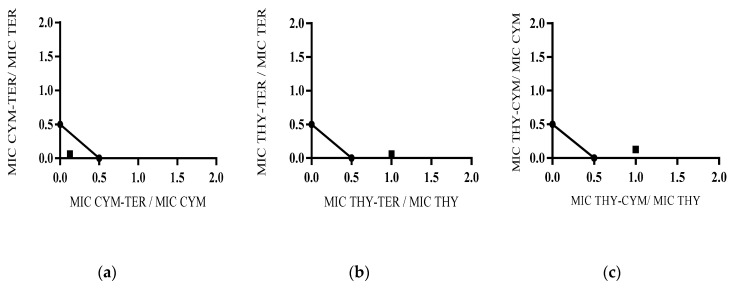
(**a**) Isobologram of p-cymene and γ-terpinene in RTO against *P. digitatum* ITEM 9569; (**b**) isobologram of thymol and γ-terpinene in RTO against *P. digitatum* ITEM 9569; (**c**) isobologram of thymol and p-cymene in RTO against *P. digitatum* ITEM 9569.

The interactions among RTOCs against *P. digitatum* ITEM 9569 were additionally evaluated in chemically reconstituted RTO using 43% thymol (25.7 µg/L), 37% p-cymene (20.0 µg/L), and 20% γ-terpinene (11.4 µg/L). The MICs of each RTOC were equal to those reported in Section 2.2.1. The MIC values of p-cymene (5.0 µg/L), and γ-terpinene (2.8 µg/L) in the ternary combination were 1/8 MIC and 1/16 MIC of the same compound tested alone, respectively. As shown in isobolograms of Figure 3a, a strong synergistic effect (FICI = 0.187) between p-cymene (FIC = 0.125) and γ-terpinene (FIC = 0.062) was found in chemically reconstituted red thyme oil diluted at 12.5% in hexane. GC-MS analysis of this ternary combination (chromatogram A in Figure S3) revealed a concentration of 0.70 ± 0.01 L/L of p-cymene (70.4 ± 0.7%), 0.29 ± 0.01 L/L of γ-terpinene (28.8 ± 0.6%), and 0.008 ± 0.001 L/L of thymol (0.78 ± 0.13%). It is interesting to note that this headspace composition is close to that found in chemically reconstituted red thyme oil diluted at 50% in hexane (Section 2.2). This result highlights that RTOCs vapors tend to spread and fill all the available volume of the High Density Polyethylene (HDPE) chamber employed for the assays and that the volatility of RTOCs is similar in both commercial and chemically reconstituted RTO. The MIC value of thymol in the THY-CYM-TER mixture was equal to that of the compound tested alone. Indeed, FICI values for THY-CYM and THY-TER combinations were above the 1.0–1.0 line, showing non-interactive effects (Figure 3b,c).

The antifungal activity of a mixture composed by the three RTOCs using ratios corresponding to the MIC values of each compound was also evaluated. This assay was carried out in order to confirm the MIC values in the ternary combination, as well as to verify the occurrence of negative interactions among RTOCs. In particular, 6.25% thymol (6.4 µg/L), 43.75% p-cymene (40.0 µg/L), and 50% γ-terpinene (45.5 µg/L) were considered. The concentration in vapor phase (chromatogram B in Figure S3) resulted 0.55 ± 0.01 L/L for p-cymene (54.9 ± 1.1%), 0.45 ± 0.01 L/L of γ-terpinene (45.0 ± 0.5%), and 0.0010 ± 0.0003 L/L of thymol (0.10 ± 0.03%). In this case, the inhibitory effect was detected only using these relative concentrations, showing non-interactive effects among RTOCs at lower concentrations (data not shown). This result confirms the MIC values of the RTOCs also in the chemically reconstituted RTO and suggests that the interactions occurring in RTO depend on the concentration of each compound and the ratios (relative quantity) among RTO volatiles.

## 3. Discussion

The antimicrobial activity of essential oils is usually determined by, or ascribed to, major compounds since it is difficult to discriminate among biological activities exerted by compounds occurring in small amounts [30]. The present work thus brings relevant data that will contribute to further studies on the antifungal activity of essential oils and to applications in several sectors where these mixtures can be employed for microbial [30] or pest [31] control. The in-silico prediction of the antifungal activity of the main RTOCs, through the linear QSAR model, showed high potential activity related to the membrane permeability disruption, even though other antifungal mechanisms were predicted at a lesser extent.

Different methods are available to evaluate the antimicrobial activity of essential oils in vapor phase. For bacteria, common methods are the use of inverted Petri dishes, polycarbonate apparatus with upper and lower chamber divided into wells and with O-rings, polycarbonate vial with upper and lower well with O-rings at the junction of the wells, and the agar plug assay [9]. However, in order to evaluate the antifungal activity of essential oils and their compounds, it is better to use hermetically sealed boxes due to slow growth rate of molds [9]. Pinto et al. (2021) used plastic boxes tightly closed with the lid and sealed with parafilm to evaluate the antifungal action of RTO against *Penicillium* spp. This method allowed to determine the antimicrobial action of RTO and to follow the concentration of RTOCs during incubation [5].

RTO showed a MIC value of 76–255 µg/mL by direct contact [32] and of 0.16–0.5 µg/mL air by vapor contact [23] against various food relevant fungi. In the present study, the mean MIC values for thymol, p-cymene and γ-terpinene were 7.0, 40.0, 28.4 µg/L, respectively. Thymol, the major compound (43%) of RTO, is an aromatic phenol that showed antifungal activity in vapor phase against longan fruit spoilage fungi with a MIC of 40 ppm [33], as well as against *Colletotrichum gloeosporioides* at a concentration lower than 5 µg/L [34]. Suwanamornlert et al. (2018) found a higher antifungal activity of thymol than carvacrol and *trans*-cinnamaldehyde in vapor phase [33]. In the present work, it was confirmed a different fungal sensitivity to thymol exposure in vapor phase. In regard to p-cymene, this monoterpene shares the carbon skeleton with thymol and carvacrol and showed no antifungal activity against fungi of the genera *Rizhopus* and *Aspergillus* [35]. Conversely, *Ciminum cymininunm* L. seed essential oil composed by 47% of p-cymene showed high antifungal activity against *A. flavus* [35]. These results support the hypothesis that p-cymene could be active interacting with other essential oil compounds of *Ciminum cymininunm* L. seed, such as γ-terpinene, cuminaldehyde, and laevo β-pinene [36]. However, limited data are available on MIC values in vapor phase against fungal strains. As concerns the antifungal action of γ-terpinene, this monoterpene significantly reduced the growth of *B. cinerea*, showing an additive effect with sabinene [37]. In our study, a MIC of 22.8 µg/L was found against *B. cinerea* ITEM 5154.

Among the three RTOCs, thymol showed the lowest MIC values, and it was responsible for the antifungal action of binary combinations with p-cymene and γ-terpinene against *B. cinerea*, *P. italicum*, *A. alternata* and *M. laxa.* In the case of *P. digitatum* ITEM 9569, thymol showed a lower antifungal activity in comparison to p-cymene, and various RTOCs combinations. Similarly, de Castro et al. (2019) found that the *Ocimum gratissimum* L. EO showed higher *Corynespora cassiicola* mycelial growth inhibition than pure thymol at the same concentration found in the aforementioned EO [38]. However, these authors did not report data on the antifungal activity of each single compound, as well as of different EOCs combinations [38]. Our results against *B. cinerea* partially confirmed those of de Castro et al. (2019), showing a better antifungal activity of the combination p-cymene and γ-terpinene in comparison with that produced by single compounds (Table 3). In *B. cinerea* and *P. digitatum*, the interactive effects were detected among the less active compound in vapor phase (p-cymene for *B. cinerea* and γ-terpinene for *P. digitatum*) (Table S1) and the other RTOCs (Table 3).

Even though thymol was the RTO compound endowed with the highest antifungal activity in vapor phase, RTO combinations without thymol still produced antifungal activity against *P. digitatum* (Table 3). These results suggest a possible combined effect of RTOCs in the antifungal activity of RTO found against this strain. For these reasons, and considering its high resistance to RTO vapors, the strain *P. digitatum* was selected as the best fungal target, among those included in this study, to verify possible interactive effects among the main RTOCs.

Possible interactive effects among RTOCs were evaluated using binary and ternary combinations. Slight synergistic interactions among thymol and γ-terpinene in binary combinations, and strong synergism between p-cymene and γ-terpinene in ternary combinations were found against *P. digitatum*. It is interesting to note that these interactions were found between an oxygenated terpene (thymol) and terpene hydrocarbons (p-cymene and γ-terpinene). Similar findings were described between several fractions of *Cymbopogon citratus*, *Ocimum gratissimum*, and *Thymus vulgaris* essential oils rich in oxygenated terpenes and terpene hydrocarbons against *P. expansum*. The authors explained synergistic interactions by the mechanism of action against the fungi: terpene hydrocarbons, such as p-cymene, could facilitate the transmembrane transportation of oxygenated terpenes, such as thymol, citral, and carvacrol [20]. According to the review of Nazzaro et al. (2017), thymol and p-cymene have multiple mechanisms of antifungal action, such as cell membrane disruption, inhibition of cell wall formation, inhibition of efflux pump, changes in mycelium morphology, and production of ROS and nitric oxide [39], whereas γ-terpinene is responsible for the protein and lipid leakage in fungi [40]. Probably, the interactions among RTOCs against *P. digitatum* could be attributed to the different mechanisms of antifungal action, as also suggested by the in-silico evaluation reported in this study.

Boubaker et al. (2016) found that *Thymus riatarum* EO, mainly composed by carvacrol (32.2%) and p-cymene (13.5%), showed the same reduction in the mycelium growth of *Geotricum citri-aurantii*, *P. digitatum*, and *P. italicum*, in comparison to *Thymus leptobotrys* EO with 76.9% of carvacrol [41]. On the other hand, *Thymus broussonnetii* subsp. *hannonis* EO, mainly composed by camphor (46.2%) and α-terpineol (7.7%), did not show antifungal effect, probably for the low concentration (<1%) of oxygenated terpenes, such as thymol and carvacrol [41]. These results suggest that the oxygenated terpenes are the main compounds responsible for the antifungal action. In order to further shed light on interactions among RTOCs in vapor phase, the antifungal action could be evaluated using different essential oils, extracted from *Thymus* species with different chemical composition.

Several EOCs have been approved for the contact with food products [27] and can be used in active packaging. However, the active EOs concentrations in vapor phase can negatively affect the sensory properties of food. This drawback can be overcome through the selection of active EOs with reduced impact on sensory properties of food [14] or the use of different application conditions in vapor phase [15]. Given the results of our work, the sensory properties of foods exposed to selected combinations of RTOCs or thyme essential oil should be further evaluated.

## 4. Materials and Methods

The experimental activity carried out in this work is reported in Figure 1.

### 4.1. Strains, Culture Conditions, and Chemicals

*Alternaria alternata* ITEM 4215, *Botrytis cinerea* ITEM 5154, *Penicillium italicum* ITEM 9571, and *Penicillium digitatum* ITEM 9569 were obtained from the Agri-Food Toxigenic Fungi Culture Collection (ITEM) of the Institute of Sciences of Food Production (Bari, Italy; http://server.ispa.cnr.it/ITEM/Collection/) and maintained on Potato Dextrose Agar (PDA, Biolife Italiana Srl, Milan, Italy) at 4 °C. *Monilinia laxa* CBS 101507 was purchased from the Westerdijk Fungal Biodiversity Institute (Utrecht, The Netherlands). Fungal strains were incubated for two weeks on PDA at 25 °C; *M. laxa* CBS 101507 cultures were incubated under light exposure, in order to promote the spore production. Spore suspensions were prepared by flooding and suspending sporified mycelium in 10 mL of sterile distilled water per plate, as well as subsequent filtration through a sterile gauze; the spore concentration was adjusted with sterile water to approximately 1.0 × 10^6^ spores/mL by using a Thoma counting chamber.

The red thyme oil (RTO, *Thymus vulgaris* L.) was purchased from Bristol Botanicals Ltd. (Bristol, UK). RTO constituents thymol (≥98.5%, THY), p-cymene (99%, CYM), and γ-terpinene (97%, TER) were obtained from Sigma-Aldrich (Merck KGaA, Darmstadt, Germany). RTOCs were diluted in n-hexane (HiPerSolv Chromanorm^®^ for High Performance Liquid Chromatography, HPLC, VWR International, Darmstadt, Germany) at 50% *v*/*v* for p-cymene and γ-terpinene or 50% *w/v* for thymol to perform antimicrobial assays. All the concentrations reported in the Results section refer to these stock solutions, freshly prepared for each assay.

### 4.2. In Silico Analysis

The major constituents of the RTO, identified by means of GC-MS analysis [5], were analyzed in silico using the program Prediction of Activity Spectra for Substances (PASS online; http://www.pharmaexpert.ru/passonline/index.php), in order to predict their potential antifungal activity. The PASS online tool performs the decomposition of the structure in descriptors and compares with the ones from biologically active compounds available in the database (with more than 250,000 compounds). The results show the probabilities of each compound to be active (Pa) and inactive (Pi). Six biological effects and five mechanisms of action were investigated for each RTOC, based on the descriptors selected by Seibert et al. (2019), for the antifungal activity [42] and others related to general antimicrobial action. The results for each of the descriptors were expressed by the difference Pa − Pi and classified as (Pa − Pi) < 0.2: low potential; 0.2 ≤ (Pa − Pi) < 0.5: moderate potential; (Pa − Pi) ≥ 0.5: high potential [42].

### 4.3. Antimicrobial Assay

#### 4.3.1. Vapor Contact Assay

Antifungal activity of the RTOCs was evaluated in vapor phase. PDA plates were covered with PT-400 cellophane membranes (Pacifici Corrado S.n.c., Rome, Italy), inoculated with 200 µL of fungal strains spore suspensions, and placed into plastic boxes in HDPE with an internal volume of 600 mL (Ref. 11673, Albero Forte Composite s.l., Banyeres de Mariola, Spain, Figure S1). Sterile discs (2 for each camera) were loaded with different volumes of thymol, p-cymene and γ-terpinene solutions in hexane (50% *v*/*v* or *w*/*v*) and placed on the inner surface of the lid. The plastic box was quickly and tightly closed with the lid, sealed with parafilm and incubated for 72 h at 25 °C.

#### 4.3.2. Determination of Minimum Inhibitory Concentration of RTOCs

In order to define the Minimum Inhibitory Concentration (MIC) of each RTOC, the composition of the RTO was considered. Preliminary tests showed that the common MIC of RTO in vapor phase was 66.6 µL/L, considering all fungal strains used in this work. This concentration was achieved using 40 µL of RTO solution in hexane (50% *v*/*v*) in a volume of 600 mL. The concentration of each compound in the plastic box was calculated taking into account the RTO composition (43% thymol, 25.7 µg/L; 37% p-cymene, 20.0 µg/L; 20% γ-terpinene, 11.4 µg/L) [5]. In order to calculate the MIC values for each RTOC against five fungal strains, thymol was added ranging from 102.9 to 1.6 µg/L, p-cymene from 80.0 to 5.0 µg/L, and γ-terpinene from 45.5 to 2.8 µg/L, using double serial dilutions in n-hexane. Inoculated plates exposed to 40 µL of n-hexane were used as controls.

At the end of incubation, cellophane membranes leading fungal biomass were carefully removed in order to measure the dry weight. The membranes were dried for 24 h at 60 °C in a fan oven. The Minimum Inhibitory Concentration (MIC) was determined as the minimum concentration that reduced the mycelium biomass of at least 20% in comparison to controls. This value was selected on the basis of variability found among different biological replicates (within the range of 10–15%).

### 4.4. Evaluation of Interactions among RTOCs

In order to exploit potential interactions among RTOCs, thymol, p-cymene, and γ-terpinene were combined using the ratios occurring in red thyme oil (43% thymol, 37% p-cymene, 20% γ-terpinene). In particular, 25.7 µg/L of thymol, 20.0 µg/L of p-cymene, and 11.4 µg/L of γ-terpinene were used, and four combinations were considered (THY-CYM-TER, THY-CYM, THY-TER and CYM-TER). The antifungal action of each combination was evaluated as described in the Section 4.4.1.

#### 4.4.1. Assessment of the FIC Index

The interactions among thymol, p-cymene, and γ-terpinene were additionally evaluated using a modified checkerboard assay [23] against *P. digitatum* ITEM 9569. Indeed, potentially interactive effects were found between p-cymene and γ-terpinene, as well as thymol and γ-terpinene only for this strain (see Results section). In order to determine CYM-TER interactions, 20 combinations were considered to evaluate the effect of these antifungal volatiles on mycelium growth. The twenty combinations were prepared in different Eppendorf tubes and then loaded onto paper discs. The concentration of p-cymene ranged from 40.0 µg/L to 5.0 µg/L, whereas the concentration of γ-terpinene ranged from 91.4 µL/L to 5.8 µg/L. As described for the MIC determination, all the controls were also included. To assess the interactions, the data obtained were further analyzed using the fractional inhibitory concentration (FIC) index, which is based on the zero-interaction theory of Loewe additivity [19]. FIC index (FICI) is defined in Equations (1) and (2) as:

FICI = FIC_CYM_ + FIC_TER_, (1)

FICI = (MIC_CYM in combination_)/(MIC_CYM tested alone_) + (MIC_TER in combination_)/(MIC_TER tested alone_). (2)

The MIC_CYM_ and MIC_TER_ are the MICs of p-cymene and γ-terpinene, respectively. A FIC index value indicated synergism with values ≤ 0.5 and antagonism with values > 4. A FIC index value between 0.5 and 1.0 was considered additive, and a value between 1.0 and 4.0 was considered as indifferent [19].

In regard to THY-TER interactions, 12 combinations were included in the assay. The concentration of thymol ranged from 25.7 µg/L to 1.6 µg/L, whereas the concentration of γ-terpinene ranged from 45.5 µg/L to 11.4 µg/L. The controls, represented by thymol, and γ-terpinene solutions were also included. To assess the interactions, the data obtained were further analyzed using the FIC index. The formula used for the FICI calculation is reported below in Equations (3) and (4).

FICI = FIC_THY_ + FIC_TER_, (3)

FICI = (MIC_THY in combination_)/(MIC_THY tested alone_) + (MIC_TER in combination_)/(MIC_TER tested alone_). (4)

#### 4.4.2. RTOCs Combinations and Isobolograms

All data were reported in isobolograms to present the MIC values of the combinations as ratios [28]. The isobolograms were interpreted by examining the data points for each ratio in relation to the MIC values for the RTO compound independently. Points below or on the 0.5:0.5 line on the isobologram were interpreted as synergistic, points between the 0.5:0.5 and 1.0:1.0 line were interpreted as additive, and points between the 1.0:1.0 line and 4.0:4.0 line were classified as non-interactive. Antagonism was identified as data points above the 4.0:4.0 line [43].

The interactions among thymol, p-cymene and γ-terpinene were additionally evaluated in chemically reconstituted red thyme oil against *P. digitatum* ITEM 9569. The three compounds were combined with ratios corresponding to the chemical composition of the commercial red thyme oil (43% of thymol, 37% of p-cymene, and 20% of γ-terpinene). The MIC of this mixture was determined. Thymol concentration ranged from 25.7 to 3.2 µg/L, p-cymene concentration ranged from 20.0 to 2.5 µg/L, and γ-terpinene from 11.4 to 1.5 µg/L.

Then, the interactions among thymol, p-cymene and γ-terpinene were evaluated combining the three RTO volatiles using the MIC of each compound (6.5 µg/L thymol, 40.0 µg/L p-cymene, and 45.5µg/L γ-terpinene); the MIC of this ternary combination was evaluated against the same fungal strain. In this case, thymol concentration ranged from 6.5 to 1.6 µg/L, p-cymene concentration ranged from 40.0 to 10.0 µg/L, and γ-terpinene from 45.5 to 11.4 µg/L. To assess the binary interactions between thymol and γ-terpinene, thymol and p-cymene, and p-cymene and γ-terpinene in RTO, the data obtained were further analyzed using the FIC values. Then, the FICI was calculated as sum of individual FIC values. The interpretation of interactions based on FICI was performed as reported in Section 4.3.1.

### 4.5. Gas Chromatography-Mass Spectrometry Analysis

Red Thyme essential oil (RTO) was subjected to GC-MS analysis using a gas chromatograph coupled to a mass spectrometer, as previously described [5]. The GC system 680 coupled to a Clarus SQ 8T mass spectrometer (Perkin Elmer) was equipped with an ELITE 5-MS (Perkin Elmer) column (0.30 m length × 0.25 mm inner diameter × 0.25 μm full thickness) and helium was used as a carrier gas at a constant pressure of 27,048 kPa ~7 psi. Detection and standard curves were achieved in electron impact mode (EI), and compounds were measured comparing peaks area of specific ions with those of the external standard purchased from Sigma-Aldrich Srl (Milan, Italy). The quantification of the three RTOCs was additionally performed in vapor phase as described in Pinto et al. (2021) [5], after 2 h from their loading into the plastic boxes. Selected combinations of thymol, p-cymene, and γ-terpinene, especially those showing interactive effects, were quantified in vapor phase.

### 4.6. Statistical Analysis

A square root arcsin transformation was applied to percentages of reduction in fungal biomass of different strains before carrying out the analysis of variance. One-way ANOVA (*p* ≤ 0.05) was used to evaluate the effect of RTOCs concentration or their combination on the percentage of reduction in the fungal biomass through the SPSS software (SPSS, Inc., Chicago, IL, USA). Multiple comparisons among individual means for each sample were made by Fisher’s least significant difference (LSD) multiple range or Tukey (HSD) test at the 95% confidence interval.

Isobolograms were plotted using GraphPad Prism, version 7 software (GraphPad Software, Inc., San Diego, CA, USA), to present the mean MIC values of the combinations as ratios.

## 5. Conclusions

In this work, the antifungal activity of red thyme oil compounds in vapor phase was evaluated. Thymol vapors showed MIC values lower than those of p-cymene and γ-terpinene against different fungal strains. It is interesting to note that, for the first time, interactive effects among the less active compounds (p-cymene for *B. cinerea* and γ-terpinene for *P. digitatum*) and the other RTOCs were found. Even though thymol was found to be the most active antifungal compound occurring in RTO, in *P. digitatum*, a synergistic effect between thymol and γ-terpinene and between p-cymene and γ-terpinene was demonstrated. Therefore, the interactive effects among RTOCs, here demonstrated for the first time in vapor phase, could lead to new strategies for the control of fungal development in foods. Selected EOC combinations, also when extracted from different vegetable sources, could be applied in food packaging, on the basis of specific food spoilage cases. Further studies should be addressed to evaluate these interactive effects in vapor phase, considering other essential oils with different chemical composition. In addition, the approach followed in this study could allow to select EOCs from different EOs, active against different microbial targets to improve the safety of goods produced by different sectors (i.e., food, pharmaceutical).

## Figures and Tables

**Table 1:** In silico test prediction of potential biological effects and potential mechanism of action of RTOCs using the Prediction of Activity Spectra for Substances (PASS) online tool.

	RTOCs ^a^		
Potential Biological Effects	THY	CYM	TER
Membrane integrity antagonist	0.769	0.698	0.510
Membrane permeability enhancer	0.517	0.514	0.459
General pump inhibitor	0.483	0.222	0.558
Antifungal	0.427	0.310	0.402
Steroid synthesis inhibitor	0.345	0.361	0.314
Oxidizing agent	0.265	0.405	0.405
Potential Mechanism of Action			
Cell wall synthesis inhibitor	0.028	0.070	0.069
DNA synthesis inhibitor	0.127	0.141	-
Protein synthesis inhibitor	0.080	-	0.161
Lanosterol 14 alpha demethylase inhibitor	0.100	0.140	0.128
Squalene epoxidase inhibitor	0.138	0.166	0.088

^a^ Values of difference between the probability to be active (Pa) and that to be inactive (Pi): (Pa − Pi). (THY) thymol; (CYM) p-cymene; (TER) γ-terpinene; (−): Not indicated or unsatisfactory. (Pa − Pi) < 0.2: low potential; 0.2 ≤ (Pa − Pi) < 0.5: moderate potential; (Pa − Pi) ≥ 0.5: high potential.

**Table 2:** Minimum inhibitory concentration (MIC) of single RTOCs (µg/L) or commercial RTO (µL/L, diluted in n-hexane at 50% *v/v*) able to produce a reduction (%) in fungal biomass higher than 20% after exposure to vapors for 72 h at 25 °C.

	MIC			
	THY	CYM	TER	RTO
*P. digitatum* ITEM 9569	6.4	20.0	45.5	66.6
*P. italicum* ITEM 9571	12.8	40.0	22.8	26.7
*B. cinerea* ITEM 5154	12.8	80.0	22.8	26.7
*A. alternata* ITEM 4215	1.6	>80.0	>45.5	26.7
*M. laxa* CBS 101507	1.6	20.0	22.8	13.3

Thymol concentrations (µg/L) assayed: 102.9, 51.4, 25.7, 12.8, 6.4, 3.2, 1.6.; p-cymene concentrations (µg/L) assayed: 80.0, 40.0, 20.0, 10.0, 5.0.; γ-terpinene concentrations (µg/L) assayed: 45.5, 22.8, 11.4, 5.7, 2.8; RTO concentrations (µL/L) assayed: 66.6, 26.7, 13.3, 6.7, 3.3.

**Table 3:** Percentages of reduction (%) in fungal biomass of different fungal strains after exposure (72 h at 25 °C) to different combinations of thymol (25.7 µg/L), p-cymene (20.0 µg/L), and γ-terpinene (11.4 µg/L).

Combination	*P. digitatum* ITEM 9569	*P. italicum* ITEM 9571	*B. cinerea* ITEM 5154	*A. alternata* ITEM 4215	*M. laxa* CBS 101507
RTO: THY-CYM-TER	100.0 ± 0.0 a	100.0 ± 0.0 a	100.0 ± 0.0 a	100.0 ± 0.0 a	100.0 ± 0.0 a
1: THY-TER	100.0 ± 0.0 a	100.0 ± 0.0 a	100.0 ± 0.0 a	100.0 ± 0.0 a	100.0 ± 0.0 a
2: THY-CYM	72.6 ± 7.9 c	100.0 ± 0.0 a	100.0 ± 0.0 a	100.0 ± 0.0 a	100.0 ± 0.0 a
3: CYM-TER	80.9 ± 8.2 b	n.d.	40.3 ± 3.6 b	n.d.	26.1 ± 2.3 c
THY	61.8 ± 4.3 d	100.0 ± 0.0 a	100.0 ± 0.0 a	100.0 ± 0.0 a	100.0 ± 0.0 a
CYM	69.5 ± 5.9 c	n.d.	n.d.	n.d.	32.3 ± 3.4 b
TER	n.d.	n.d.	n.d.	n.d.	n.d.

n.d.: not detected (growth not different from control); One way-ANOVA was applied to estimate the effect of different essential oil compound (EOC) combinations on fungal biomass reduction; the least significant difference (LSD) values (*p* ≤ 0.05) were calculated to separate mean values for each strain: *P. digitatum*, 3.3%; *B. cinerea*, 2.8%; *M. laxa*, 0.9%; *P. italicum* and *A. alternata*, 0%. Mean values with different lowercase letters differ significantly (*p* ≤ 0.05).

## Supplementary Materials

The following are available online. Table S1: Minimum Inhibitory concentration of RTOCs against fungal strains, Figure S1: plastic box used for antifungal assays, Figure S2: chemical composition of commercial RTO, Figure S3: representative chromatograms of RTOCs combinations.
