# Supplementary material for: Unravelling the Antifungal Effect of Red Thyme Oil (Thymus vulgaris L.) Compounds in Vapor Phase
Source: Molecules. 2020 Oct 16;25(20):4761. doi: 10.3390/molecules25204761 (PMC7587587; doi:10.3390/molecules25204761)
Supplement: Supplementary file 1 [file molecules-25-04761-s001.pdf]

molecules

Supplementary results

Table S1. Percentages of reduction (%) in fungal biomass of different strains after exposure (72 h at 25 °C) to different concentrations of thymol, p-cymene and ɣ-terpinene diluted in n-hexane at 50% (*w*/*v* or *v*/*v*).

	Concentration (µg/L)	Percentage of fungal biomass reduction (%)				
		P. digitatum ITEM 9569	*P. italicum* ITEM 9571	*B. cinerea* ITEM 5154	*A. alternata* ITEM 4215	*M. laxa* CBS 101507
**A**						
Thymol	102.9	100.0 ± 0.0a	100.0 ± 0.0a	100.0 ± 0.0a	100.0 ± 0.0a	91.9 ± 7.4a
	51.4	100.0 ± 0.0a	100.0 ± 0.0a	100.0 ± 0.0a	100.0 ± 0.0a	77.7 ± 10.2b
	25.7	60.1 ± 1.1b	100.0 ± 0.0a	100.0 ± 0.0a	100.0 ± 0.0a	62.0 ± 8.7b
	12.8	59.0 ± 3.b	74.5 ± 10.2b	100.0 ± 0.0a	100.0 ± 0.0a	54.3 ± 7.3c
	6.4	42.4 ± 2.1c	n.d.	n.d.	91.3 ± 8.3a	40.0 ± 5.4c
	3.2	10.2 ± 1.3d	n.d.	n.d.	89.2 ± 8.6a	35.5 ± 3.9c
	1.6	9.6 ± 1.4d	n.d.	n.d.	86.3 ± 10.3a	37.5 ± 3.8c
	MIC (µg/L)	6.4	12.8	12.8	1.6	1.6
**B**						
p-cymene	80.0	63.4 ± 2.5a	46.8 ± 6.1a	100.0 ± 0.0	15.2 ± 1.1	100.0 ± 0.0a
	40.0	69.3 ± 2.4a	46.8 ± 5.3a	n.d.	n.d	100.0 ± 0.0a
	20.0	28.7 ± 3.2b	n.d.	n.d	n.d.	100.0 ± 0.0a
	10.0	n.d.	n.d.	n.d.	n.d.	n.d
	5.0	n.d.	n.d.	n.d.	n.d.	n.d
	MIC (µg/L)	20.0	40.0	80.0	-	20.0
**C**						
ɣ-terpinene	45.5	100.0 ± 0.0a	100.0 ± 0.0a	100.0 ± 0.0a	10.8 ± 1.5a	100.0 ± 0.0a
	22.8	n.d.	62.5 ± 7.9b	77.8 ± 8.9b	12.3 ± 1.8a	100.0 ± 0.0a
	11.4	n.d.	n.d	10.6 ± 2.9c	9.4 ± 1.2a	n.d
	5.7	n.d.	n.d	13.1 ± 1.4c	n.d.	n.d.
	2.8	n.d.	n.d.	11.1 ± 1.7c	n.d.	n.d.
	MIC (µg/L)	45.5	22.8	22.8	-	22.8

n.d.: not detected (growth not different from control); One way- ANOVA was applied to estimate the effect of thymol concentration on fungal biomass reduction; the least significant difference post-hoc test ($p \leq 0.05$) was applied to separate mean values for each strain. Mean values with different lowercase letters differ significantly ($p \leq 0.05$).

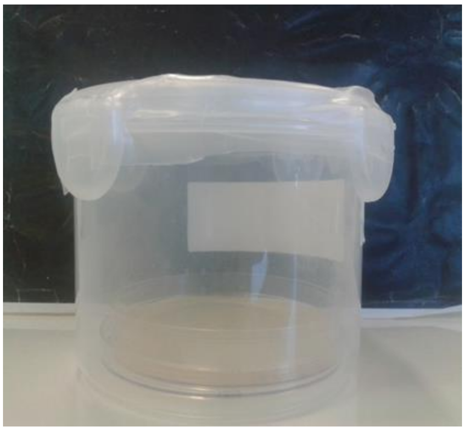

Figure 1. Plastic box of 600 mL used to evaluate the antifungal action of RTOCs in vapour phase.

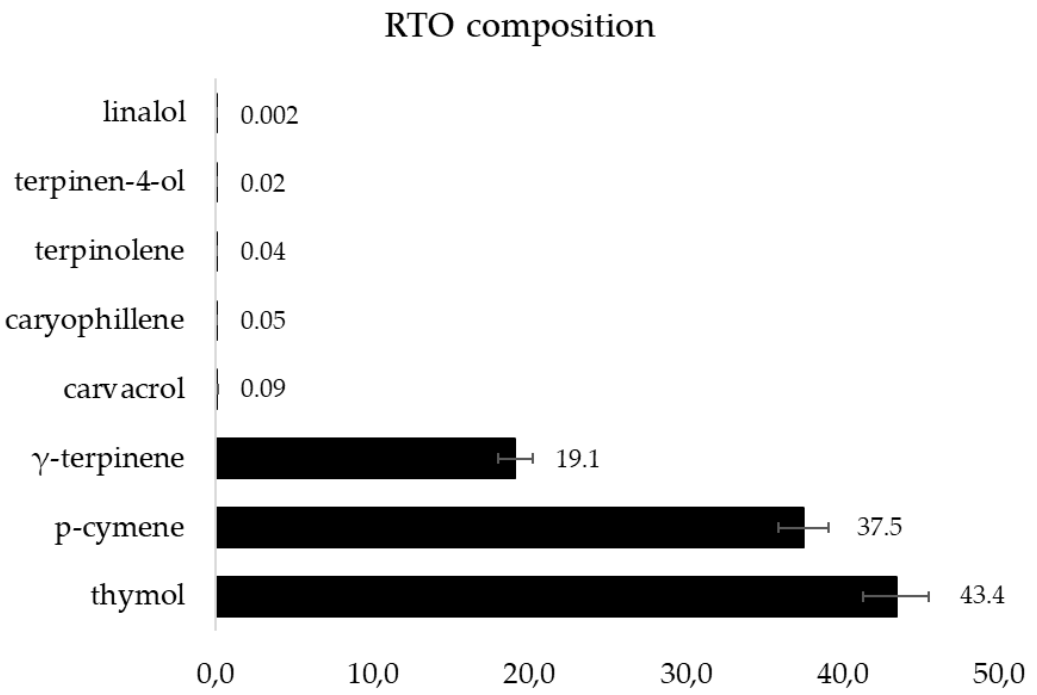

Figure S2. Chemical composition (expressed as % of each compound) of liquid RTO achieved through GC-MS analysis.

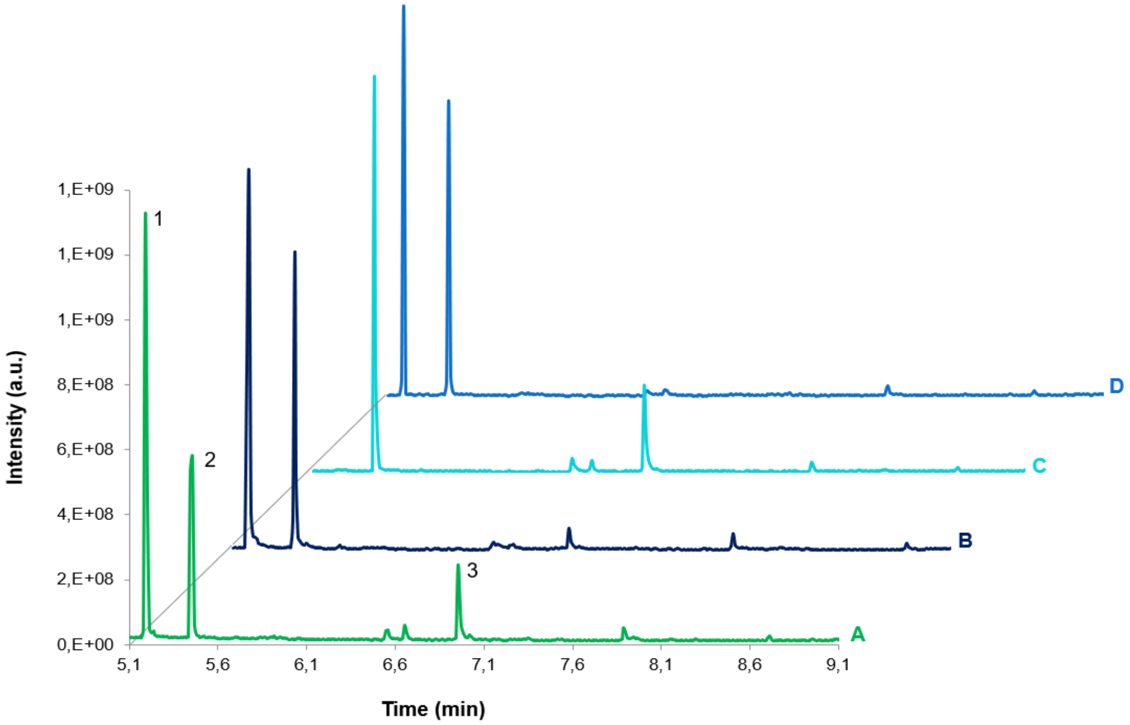

Figure S3. Representative chromatograms of samples. Peak 1: p-cymene, peak 2: ɣ-terpinene, peak 3: thymol. A: p-cymene 0.70 ± 0.01 L/L, ɣ-terpinene 0.29 ± 0.01 L/L and thymol 0.008 ± 0.002 L/L. B: p-cymene 0.55 ± 0.01 L/L, ɣ-terpinene 0.45 ± 0.01 L/L and thymol 0.0010 ± 0.0003 L/L. C: ɣ-terpinene 0.990 ± 0.030 L/L and thymol 0.013 ± 0.003 L/L. D: p-cymene 0.56 ± 0.02 L/L and ɣ-terpinene 0.44 ± 0.01 L/L.
